# Development and Application of a New QuEChERS Method in UHPLC-QqQ-MS/MS to Detect Seven Biogenic Amines in Chinese Wines

**DOI:** 10.3390/foods8110552

**Published:** 2019-11-05

**Authors:** Shun-Yu Han, Lan-Lan Hao, Xiao Shi, Jian-Ming Niu, Bo Zhang

**Affiliations:** Gansu Key Laboratory of Viticulture and Enology, College of Food Science and Engineering, Gansu Agricultural University, Lanzhou 730070, China; hanshunyu@gsau.edu.cn (S.-Y.H.); haoll@st.gsau.edu.cn (L.-L.H.); shix@st.gsau.edu.cn (X.S.); niujm@st.gsau.edu.cn (J.-M.N.)

**Keywords:** biogenic amines, wine, UHPLC-QqQ-MS/MS, modified QuEChERS, hexi corridor region, correlation analysis

## Abstract

The aim of this study was to develop and validate an improved, simple, and sensitive method for the simultaneous determination of seven types (cadaverine, CAD; hexylamine, HEX; histamine, HIS; phenylethylamine, PEA; putrescine, PUT; tyramine, TYR) of biogenic amines (BAs) in wine matrices. For this reason, a modified QuEChERS combined with ultra-performance liquid chromatography coupled to a triple quadrupole mass spectrometry (UHPLC-QqQ-MS/MS) method was investigated. The optimization of UHPLC-QqQ-MS/MS separation and QuEChERS procedure was performed. Under optimum conditions, the excellent chromatographic performance of the whole separation was accomplished within 6.3 min analyzing time. Meanwhile, the recoveries ranged from 77.2% to 101.7%, while relative standard deviation (RSD) remained between 0.0% and 9.4%. The limit of detection (LOD, 0.50–1.00 µg/L) and the limit of quantification (LOQ, 1.65–3.30 µg/L) were lower than those permitted by legislation in food matrices, which demonstrated the high sensitivity and applicability of this efficient method. This validated method was also applied in a pilot study to analyze BAs in 81 wine samples from Hexi Corridor Region (Gansu Province, Northwest China), CAD, HEX, HIS, PEA, PUT, and TYR were detected to varying degrees in the samples. However, when compared with the existing standards, the BAs in all 81 wine samples did not exceed the prescribed limit value or toxic dose (2–40 mg/L). Moreover, a statistical approach was also conducted using Pearson correlation analysis, and to evaluate their concentrations in terms of wine parameters (storage time, grape variety, wine type, and basic physicochemical index). The results showed that, among the seven kinds of BAs, the concentration of HIS had a certain correlation with alcoholic degree and grape variety. In addition, the level of PEA had a certain correlation with the wine pH and wine storage time. It is worth noting that this seems to be the first report regarding the application of QuEChERS-UHPLC-QqQ-MS/MS in the analysis of BAs in wine in this region.

## 1. Introduction

Biogenic amines (BAs) are toxic nitrogenous organic compounds that are mainly produced by microbial decarboxylation of free amino acids. Previous research found that the BAs appeared in a wide variety of food, such as meat [1] and fishery products [2]. Potentially, BAs also found in alcoholic beverages, all the microbial groups that participate in the winemaking process may be associated with BA production. In addition, there is general agreement that yeasts make a less significant contribution than lactic acid bacteria to the final concentration of BAs in wine [3]. By their structure, BAs can be divided into three well-defined categories, which are aliphatic, aromatic, and heterocyclic amines [4].

BAs serve as an important component in organisms and play a key role in maintaining the basic physiological functions of biological cells. For example, a small amount of BAs is able to control the activity of the nervous system, eliminate free radicals, regulate blood pressure, enhance metabolic activity, and intestinal immunological competence [3]. Nevertheless, an excessively high dosage of such substances can lead to nausea, headache, unstable blood pressure, and other symptoms, thus causing harm to human health [3]. For this reason, many countries have established monitoring programs and legislation to control the BAs in food. The US government (FDA) requires that tyramine (TYR) and histamine (HIS) in food should be less than 100 mg/Kg and 500 mg/Kg, respectively [5]. The EU stipulates that the concentration of HIS and TYR in food should be 100 mg/Kg and 100–800 mg/Kg at most, respectively [6]. Meanwhile, the European Food Safety Authority (EFSA) has also released a research report on BAs in fermented food and clearly explained the toxicity of them [7]. Especially, fermented liquors have attracted more attention because they contain ethanol and a traceable amount of acetaldehyde, which can significantly enhance the toxicity of BAs [8].

Given the seriousness and urgency of the above problems, higher requirements have been placed on the BA determination methods and techniques. However, the analytical determination of BAs, both individually and simultaneously, is not an easy task because of their structure and generally low concentrations present in complex matrices. Many techniques have been developed to quantify these compounds in food products, including gas chromatography-mass spectrometry (GC-MS) [9], ion chromatography (IC) [10], enzymatic methods, and immunoassays [11]. Due to its high sensitivity, resolution and versatility, high performance liquid chromatography (HPLC) with fluorescence or ultraviolet detector is the most extensively used technique for the determination of BAs in foods [12]. However, studies have shown that BA molecules involve no fluorescence, ultraviolet absorption, or electrochemical activities. Therefore, the commonly used detecting method is derivatization technology [13]. However, this operation is not only time-consuming and laborious but also increases the consumption of reagents. Hence, in the last few years, high performance liquid chromatography coupled with tandem mass spectrometry (HPLC-MS/MS) and ultra-performance liquid chromatography coupled to a quadrupole-time of flight mass spectrometry (UPLC/Q-TOFMS) have been proposed to increase the performance of BA analysis without the derivatization process [14].

Additionally, in order to minimize the loss of target analytes and subsequent decrease of recoveries, a lot of effort has been made to develop and standardize techniques of sample preparation. Traditional liquid–liquid extraction (LLE) [15], dispersive liquid–liquid microextraction (DLLME) [16], solid phase extraction (SPE) [17], and immersion solid phase microextraction (DI-SPME) [18] are commonly used techniques as pretreatment procedures. Meanwhile, researchers also developed the dispersive solid phase extraction technology, which to a large degree solved the problems that occurred where traditional analysis methods were applied, such as long sample pretreatment time, the large usage amount of toxic organic solvents, and the substances that can easily interfere with the qualitative and quantitative results. Since the technology possesses the characteristics of Quick, Easy, Cheap, Effective, Rugged, and Safe, it is named QuEChERS [19].

The Hexi Corridor Region (Gansu Province, Northwest China) is a historic and major wine production area in China. The dry and cold climate throughout the year guarantees a reduction in pests and diseases in the process of grape cultivation. Therefore, it has also been known as the major production area of organic grapes and wines in China. In order to improve the overall quality of local wine, it is necessary to ensure the basic safety of wine production. The aim of the study was to develop QuEChERS procedures and the rapid UHPLC-QqQ-MS/MS method to carry out the simultaneous determination of seven common BAs (hexylamine (HEX), tryptamine (TRY), HIS, TYR, PUT, CAD, and PEA) in wines. Meanwhile, an additional goal was to apply the optimized and validated method to wine samples from the local wineries. As far as we know, this study can provide the first report regarding the application of QuEChERS-UHPLC-QqQ-MS/MS in BA analysis of wine in this region.

## 2. Materials and Methods 

### 2.1. Chemicals and Standards

The BA reference standards of CAD, HEX, HIS, PEA, PUT, TRY and TYR were purchased from Dr. Ehrenstorfer (purity higher than 99%, Augsburg, Germany). 1,7-diaminoheptane (DAH, purity, 98%) was obtained from Pu Tian Tong chuang Biotechnology Co., Ltd. (Beijing, China). HPLC grade methanol, acetonitrile, and formic acid were from Merck KGaA (Darmstadt, Germany), analytical grade trichloromethane, butanol, ethyl acetate, anhydrous sodium carbonate (Na_2_CO_3_), and sodium chloride (NaCl) were from Dengfeng Chemical Co., Ltd. (Tianjin, China). Distilled water was obtained from Watsons (Guangzhou, China). Octadecylsilane (C18) sorbent and primary secondary amine (PSA) were acquired from Agilent Technologies (Waldbronn, Germany).

The individual stock solutions of CAD, HEX, HIS, PEA, PUT, TRY, TYR, and DAH at 100 mg/L were prepared in distilled water. A working mixed standard solution at 1 mg/L and internal standard solution at 100 mg/L were prepared immediately before use by diluting the individual stock solution in 0.1% formic acid-water solution (*v*/*v*). The standard solutions were stored under refrigerator conditions (4 °C) and protected from light. Prior to use, the working mixed standard solution was further diluted with 0.1% formic acid-water solution (*v*/*v*) to obtain the specific concentration required for the experiment.

### 2.2. Optimization of the Extraction and Instrument Method

**Figure 1:**
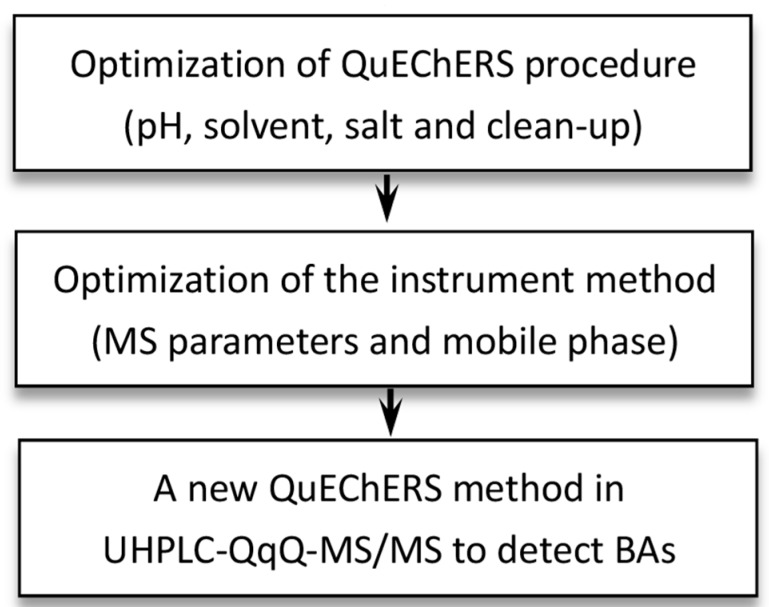
The scheme of the optimization procedure.

To obtain the simple and effective method for the extraction and analysis of major BAs in wines, a range of factors that might influence the efficiency of the method were carefully investigated. The specific scheme of analytical procedure is shown in Figure 1.

#### 2.2.1. Optimization of the Extraction Method

##### The pH Optimization

In order to inhibit the nonionic dissociation of BAs, 5 levels of pH were selected, including 3.6, 6.0, 8.0, 10.0, and 12.0. The peak intensities of the test analytes corresponding to different pH values were estimated.

##### The Extraction Solvent Optimization

Under the condition of adjusting the pH value of sample solution to 12, the extraction effects of acetonitrile, ethyl acetate, trichloromethane-butanol (1:1, *v*/*v*), and 10% methanol-acetonitrile (*v*/*v*) were compared. The peak intensities and recoveries of seven kinds of BAs were used as indexes to determine the extraction solvent by a series of experiments.

##### The Amount and Type of Salt Optimization

Under the optimal pH and extraction solvent determined above, the sole additive amount of NaCl and Na_2_CO_3_ (3 g, respectively), and the combined additive amount of NaCl and Na_2_CO_3_ (1.0 g NaCl + 2.0 g Na_2_CO_3_, 2.0 g NaCl + 1.0 g Na_2_CO_3_,) were optimized. The recoveries of seven kinds of BAs were used as indexes to determine the optimal added salt amount and salt types by a series of experiments.

##### The Clean-Up Optimization

In order to improve recoveries provided by the aforementioned procedure, various dSPE sorbents (C18, PSA) and sorbent mixtures (C18 + PSA) were tested during the clean-up step, respectively.

##### Optimized QuEChERS Procedure

Lastly, the optimized QuEChERS extraction procedure was employed to extract BAs from the wine samples. Briefly, 2.0 mL of sample was added to a 50 mL centrifuge tube with 30 μL DAH and 5 mL distilled water. The pH of the solution was adjusted to 12 by adding NaOH and homogenized on vortex mixer (Scientific Industries. Inc, USA) for 2 min. Then, 5 mL of trichloromethane-butanol (1:1, *v*/*v*), 1 g NaCl and 2 g Na_2_CO_3_ were added, and the tubes were shaken for 5 min at room temperature. After extraction, the tubes were centrifuged for 5 min (10,000 rpm) with a 3K30 centrifuge (Sigma-Aldrich, Inc, (Steinheim, Germany)). The upper organic phase was transferred into a 15 mL vial and this extraction step was repeated. The extracted solution was diluted by 10 mL trichloromethane-butanol (1:1, *v*/*v*). Aliquots of 1 mL above solution containing 100 μL formic acid was evaporated under a gentle stream of nitrogen and reconstituted to 1 mL with 0.1% formic acid-water solution (*v*/*v*) for analysis. The extracts were stored at 4 °C until analysis in order to preserve their stability. Prior to injection, the sample was filtered through a 0.22 μm polyvinylidene fluoride (PVDF) filter (Anpu Scientific Instruments Co., Ltd., (Shanghai, China)). 

#### 2.2.2. Optimization of the Instrument Method

Chromatographic analyses were performed in an Agilent 1290 Infinity LC Series coupled to a 6460 Triple Quadrupole (QqQ) mass spectrometer with an electrospray ionisation (ESI) interface, all from Agilent Technologies (Waldbronn, Germany), operating in positive ion mode. Chromatographic separation was performed using an Eclipse Plus C18 (100 mm × 2.1 mm, 1.8 μm) from Agilent Technologies (Waldbronn, Germany). In addition, 0.1% formic acid-water solution (eluent A) and 0.1% formic acid-acetonitrile solution (eluent B) were used as the mobile phase at a constant flow of 0.2 mL/min. Column temperature was set at 30 °C and the injection volume was 2 µL.

MS/MS analyses of BAs were performed on the 6460 QqQ mass spectrometer with Agilent Jet Stream Technology under the dynamic multiple reaction monitoring (DMRM) conditions in ESI^+^. The following settings were used: nebulizer, 45 psi; drying gas temperature, 300 °C; drying gas flow, 10 L/min; capillary voltage, 4000 V. Fragmentor voltage and collision energies were optimized for each analyte during infusion of the pure standard, and the most abundant fragment ion was chosen for the selected reaction monitoring (Table 1).

### 2.3. Method Validation

Performance characteristics of the optimized method were established by a validation procedure with samples, studying linearity, LODs and LOQs, precision, and recovery.

The linearity calibration standards were prepared by combining a series of mixed working standard solutions into the blank wine samples to yield the desired concentrations in the range of 0.5 µg/L to 800 µg/L for each analyte (*n* = 3), and DAH concentration was 300 μg/L. Calculation curves were performed based on the average peak areas by internal standard calibration. LODs and LOQs were calculated as produce chromatographic peak at signal-to-noise ratio (S/N) of 3 and 10, respectively.

Precision was evaluated studying intraday (repeatability) and interday precision, and they were expressed as relative standard deviation (RSD). For intraday precision, results were obtained from the injection of six spiked samples at concentration levels of 50, 100 and 500 μg/L, while for interday precision, three spiked samples, at the same aforementioned concentration levels, were analysed in five different days.

Recovery studies were performed by spiking blank wine samples with the corresponding volume of the multi-compound working standard solutions (with 300 μg/L of DAH). Three concentrations were evaluated (50, 100 and 500 μg/L) by spiking three blank wine samples at each concentration level, and the pretreatment and instrumental parameters of samples are consistent before and after spiking. 

### 2.4. Sample Preparation

Wine samples were collected in local wineries (from 3 different geographic areas of Hexi Corridor Region, which are Jiayuguan, Zhangye and Wuwei, respectively.) and supermarkets. Eighty-one wine samples, which include 15 commercial brands, were selected during the years 2008–2017 from various wineries. At least 3 L/sample were collected, and all samples were stored in the dark at 4 °C before analysis. 

### 2.5. Data Analysis

BA identification and quantitation analyses in wine samples were performed using Agilent’s Mass Hunter Quantitative Analysis Software (version B.07.00). Microsoft Excel 2010 (Microsoft, Redmond, WA, USA) was used to calculate the mean, standard deviation and RSD. Statistical significance of the data and Pearson correlation analysis were performed using SPSS 19.0 (SPSS Inc., Chicago, IL, USA). Data was subjected to one-way analysis of variance (ANOVA) and multiple comparative analysis was performed on Duncan’s test.

## 3. Results

### 3.1. Optimization of the UHPLC-QqQ-MS/MS Conditions 

Before the optimization of the separation efficiency of the chromatographic system, the choice of mobile phase should firstly focus on the ionization efficiency before the analytes enter the MS/MS system. With the aim to obtain high sensitivity, a standard solution of 1 mg/L of each analyte was individually infused directly into the MS/MS system and analyzed in both positive and negative ion modes carrying out MS full scan. The results showed that all analytes could generate [M+H]^+^ precursor ions under the ESI^+^ mode. Then, dissociation with nitrogen was induced, and different collision energies were tested to identify the most abundant product ion scanned in the DMRM mode (Table 1).

**Figure 2:**
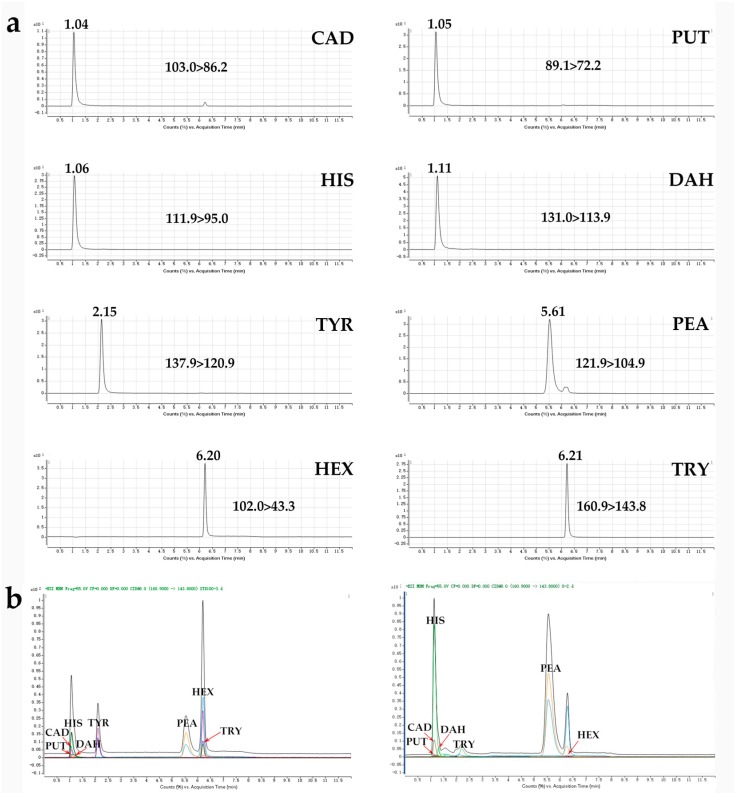
The extracted ion chromatograms of seven biogenic amines (BAs) and the internal standard in a standard solution under positive mode (**a**), and total ion chromatograms and extracted overlaid dynamic multiple reaction monitoring (DMRM) chromatograms obtained from ultra-performance liquid chromatography coupled to a triple quadrupole mass spectrometry (UHPLC-QqQ-MS/MS) analysis in full-scan mode (**b**), (left) standard solution; (right) real wine sample (CAD: Cadaverine; PUT: Putrescine; HIS: Histamine; DAH: 1,7-diaminoheptane; TYR: Tyramine; PEA: Phenylethylamine; HEX: Hexylamine; TRY: Tryptamine).

Meanwhile, a suitable solvent system is also a key parameter in separation. As for the choice of the elution mobile phase, methanol and acetonitrile were considered. The results showed that when methanol-water was used as the mobile phase, a large interference peak often appeared, and the target peaks of the seven kinds of BAs over lapped severely and could not take shape separately. In contrast, the usage of acetonitrile resulted in much better separation of the analytes and all of them were stable. This may be attributed to the high solvent strength and low viscosity of acetonitrile [20]. Furthermore, considering that BA belongs to an organic base, some of its components present certain alkalinity and may create the tailing phenomenon in analysis [21]. Therefore, in order to improve the separation efficiency of BAs, the addition of 0.1% formic acids (*v*/*v*) added to the mobile phase was tested. It was found that when a small amount of formic acid was added to the acetonitrile-water system, the separation between the target objects became more complete, and the peak response value increased significantly (Figure 2a). This is mainly because a trace amount of formic acid in the mobile phase system is able to lower the activity of silicon hydroxyl in the chromatographic column [22], which is conducive to the formation of good peak shape of the target objects, and to enhance the responses of [M+H]^+^ ions, thereby improving detection sensitivity.

In addition, in order to separate the multiple BA components simultaneously in a short period of time and with good peak shape, optimization was done based on a linear gradient elution program. This method can measure seven kinds of BAs within 6.3 min, and finish a complete sample injection within only 12.0 min (Figure 2b). In addition, the peak shape is good and the degree of separation is high, so it is suitable for large-batch detection.

### 3.2. Optimization of the QuEChERS Procedure

**Figure 3:**
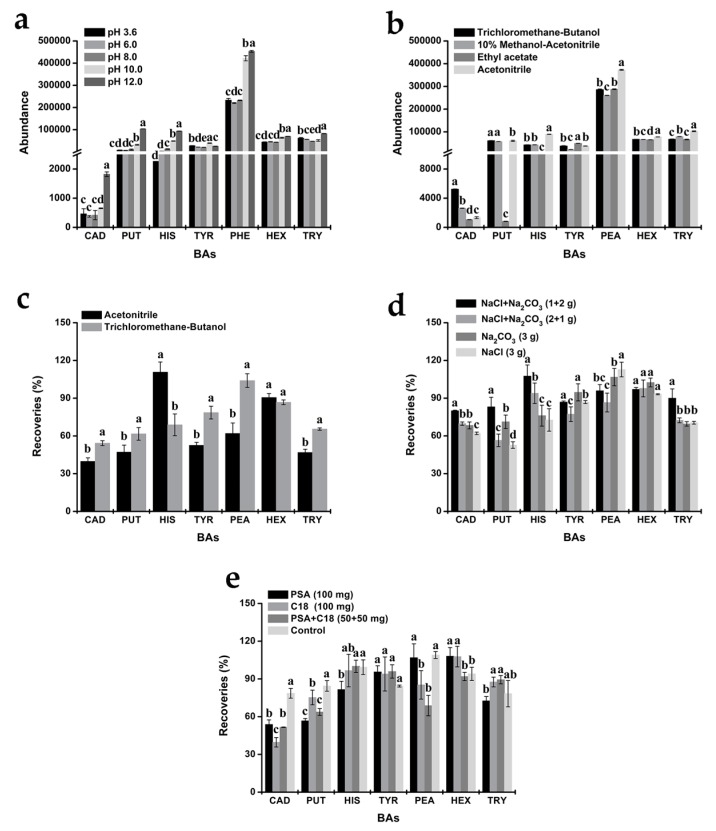
Abundance and recoveries obtained by different QuEChERS procedures (**a**). the pH optimization; (**b**). the extraction solvent optimization; (**c**). the recovery percentage obtained by applying acetonitrile and trichloromethane-butanol solutions; (**d**). the amount and type of salt optimization; (**e**). the clean-up optimization; different letters in same BA are statistically significant *p* ˂ 0.05; CAD: Cadaverine; PUT: Putrescine; HIS: Histamine; TYR: Tyramine; PEA: Phenylethylamine; HEX: Hexylamine; TRY: Tryptamine).

BA molecule is a kind of organic base compound, whose non-ionic dissociation can be inhibited under alkaline conditions. Therefore, BA presents the molecular structure, which is conducive to solvent extraction. In this experiment, NaOH was added to adjust the pH of the solution, and the peak intensity of the analytes when pH was set between 3.6 and 12.0 was investigated. The results showed that the peak intensity of the seven kinds of BAs was low when pH was between 3.6 and 6.0. When pH was between 8.0 and 12.0, the peak intensity increased gradually (Figure 3a), which was in agreement with the previous result [23], and revealed the importance of accurately controlling and adjusting sample’s pH before the extraction takes place, because it could greatly influence the recovery of amines. Finally, the peak abundance was the highest when the pH was at 12.0, and the difference was significant compared with that of other pH conditions. Therefore, pH 12.0 was selected as the most appropriate pH level for further study.

An ideal sample pretreatment way is to use a simple extraction method to distribute the target objects in the extracting solution as much as possible. The extraction effects of acetonitrile, ethyl acetate, trichloromethane-butanol (1:1, *v*/*v*), and 10% methanol-acetonitrile (*v*/*v*) were compared in this experiment. By comparing the peak abundance of the seven kinds of BAs, it was found that acetonitrile and trichloromethane-butanol solutions demonstrated better overall extraction efficiency for seven BAs, which assumed significant difference from other extraction agents (Figure 3b). Meanwhile, on the basis of the above optimized conditions, the recoveries of the seven BAs by acetonitrile and trichloromethane-butanol were further measured. It could be clearly observed from Figure 3c that the recovery ratio of trichloromethane-butanol on these BAs (ranged from 54.25% to 104.01%, and the average recovery ratio reached 74.21%) were higher than those acetonitrile. Therefore, trichloromethane-butanol is the optimal extraction solvent when the QuEChERS method is applied.

In order to overcome the emulsification in the extraction process and promote the separation of organic and aqueous phases, the influence of NaCl and anhydrous Na_2_CO_3_ as salting-out agents on the extraction was investigated experimentally, and the amount of both agents was determined (Figure 3d). The results indicated that when NaCl or Na_2_CO_3_ was used alone, trichloromethane-butanol solvent had no significant effect on the extraction of the seven kinds of BAs in the wine and exerted no assisting effect on the extraction. When these two kinds of salt were added together, the recovery ratio of these BAs was significantly higher, and the extraction effect was more pronounced when the amount of Na_2_CO_3_ increased. By adjusting the dosage of NaCl and anhydrous Na_2_CO_3_, it was found in the experiment that 1 g NaCl and 2 g Na_2_CO_3_ could achieve a higher recovery ratio than that achieved by other combinations of salt dosage.

In order to further reduce the possible matrix effect in the extraction and detection processes, and to improve the detection accuracy, the purifying effects of C18, PSA and C18+PSA as adsorbent were investigated. The results showed that the purifying effects of these substances were significantly different and would affect the recovery ratio of PUT and CAD to varying degrees (Figure 3e). In addition, the layering between the extraction agent and PSA was not obvious, thus it was easy to form a turbid solution. However, from the comparison of the recovery ratio between the target objects treated by cleaning agent and those undergone sample injection after direct nitrogen blowing and volume metering, it can be seen that the recovery ratio of the seven kinds of BAs that did not undergo purification treatment satisfied the method validation requirements. When it comes to the purified samples, although five kinds of BAs had a good recovery ratio, the recoveries of PUT and CAD werelow (below relevant method validation standards). The statistical comparison showed that there was no significant difference in terms of the recovery ratio of BAs between purified samples and non-purified ones (*p* > 0.05). Even though non-purified treatment increased the replacement of nitrogen blowing solvent, it reduced the use of adsorbent and saved the treatment time. Therefore, after overall consideration, non-purification treatment was finally chosen as the optimized QuEChERS scheme.

### 3.3. Method Validation

In terms of method validation, linear range, LOD, LOQ, precision, and recovery were studied, respectively. Good linear relationships were achieved with linear regression coefficients (*R*^2^) higher than 0.9900 over the examined concentration range as showed in Table 2. Under the optimized conditions, the LOD and LOQ of the test analytes were 0.50–1.00 µg/L and 1.65–3.30 µg/L, respectively. The precision of the method was determined by analyzing wine samples which contain all BAs at concentration levels ranging from 50 µg/L to 500 µg/L, and calculating the concentrations from external calibration. Data in Table 2 can show that recoveries ranging from 77.18% to 101.69% of all tested BAs with RSD are lower than 10%, which indicated that the proposed QuEChERS method coupled with UHPLC-QqQ-MS/MS analysis was sufficiently accurate for the determination of the analyte concentrations in the wine samples.

### 3.4. Application to Wine Samples

In addition, 81 commercially available wine samples from a number of wineries of the Hexi Corridor Region were analyzed (Table S1). In total, six kinds of BAs were detected, including CAD, PUT, HIS, TYR, PEA, and HEX. The results showed that 39 out of 81 samples (about 48% of incidence) contaminated all above mentioned BAs, ranging from 728.14 to 12,493.55 μg/L. The relative concentrations of the six BAs followed the order: PUT (mean = 2905.96 μg/L and median = 3297.93 μg/L) > HIS (mean = 839.97 μg/L and median = 88.10 μg/L) > TYR (mean = 312.82 μg/L and median = 25.10 μg/L) > PEA (mean = 453.65 μg/L and median = 302.03 μg/L) > CAD (mean = 240.94 μg/L and median = 133.43 μg/L) > HEX (mean = 27.04 μg/L and median = 24.32 μg/L).

**Figure 4:**
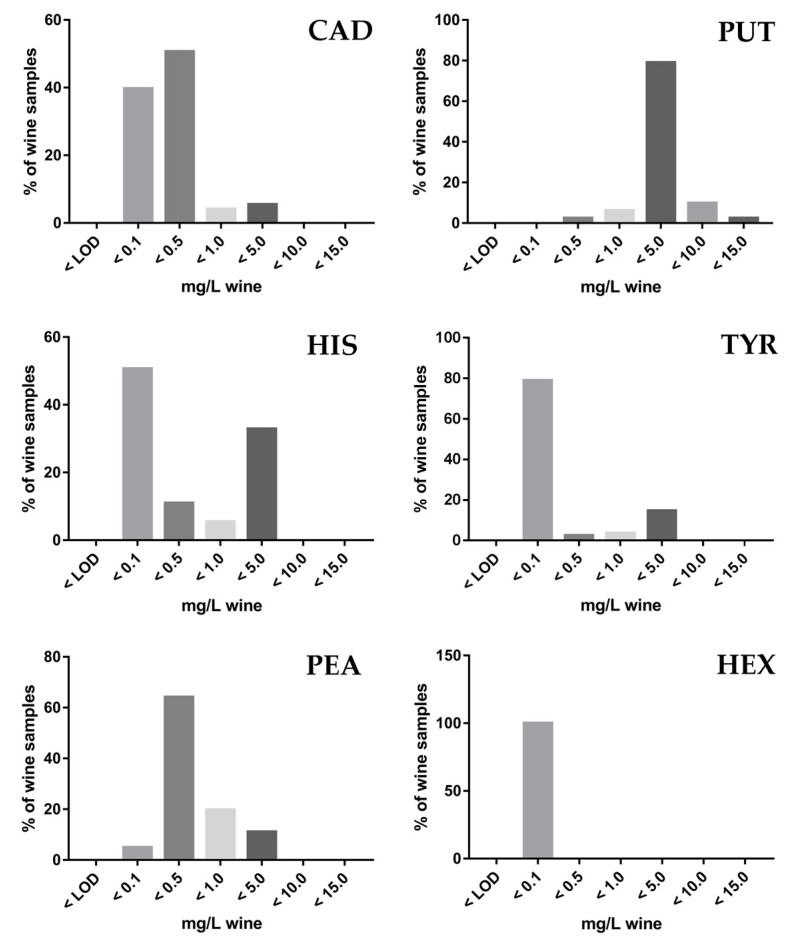
Relative distribution of all tested wine samples analyzed in this study referring to their BA concentrations (CAD: Cadaverine; PUT: Putrescine; HIS: Histamine; TYR: Tyramine; PEA: Phenylethylamine; HEX: Hexylamine).

In order to understand the actual distribution of the samples, the relative distribution of all analyzed wines was presented based on their BA contents (Figure 4). It is obvious that few samples had high BA contents (>10,000 μg/L wine) except PUT. In particular, some individual samples showed very high levels of it (e.g., two kinds of dry red wines with 10,349.91 and 10,787.44 μg/L). For most of the wines (>62%), we found that the individual BA was generally present at concentrations <1000 μg/L. Although there was a wide variation in the amount of these substances, considering the toxic dose of BA in alcoholic beverages which varies between 8 and 20 mg/L for HIS, between 25 and 40 mg/L for TYR, and 3 mg/L for PEA [5], none of the examined sample exceeds the specified toxic doses of these compounds. However, it should be noted that, for the 81 wine samples, 73 (90%) were contaminated with both of the BAs (Table S1). Such co-contamination was most evident among red ice wines, dry red wines, and semi-sweet red wines in all samples (95% of red ice wines, 97% of dry red wines, and 100% of semi-sweet red wines). Co-contamination was also commonly observed in dry white wines and white ice wines in all samples (100% of dry white wines and white ice wines).

To obtain a better understanding of the kind and concentration of BAs in different types of wine, BA occurrences were summarized in Table 3. PUT, TYR, and PEA were found in all 81 wine products, with the highest mean and maximum levels found in dry red wines and white ice wines, respectively (3525.31 and 10,787.44 μg/L for PUT; 470.49 and 2807.30 μg/L for TYR; 633.86 and 2378.34 μg/L for PEA). In addition, 73 out of 81 samples (90%) were contaminated with CAD and HIS, respectively (CAD: 67% of dry white wines and sweet red wines, 83% of white ice wines, 92% of dry red wines, and 100% of red ice wines and semi-sweet red wines; HIS: 33% of sweet red wines, 50% of white ice wines, 67% of dry white wines, 97% of dry red wines, and 100% of red ice wines and semi-sweet red wines). In addition, 45 samples (56%) were contaminated with HEX (33% of dry white wines, 50% of white ice wines and red ice wines, 56% of dry red wines, 67% of sweet red wines and 100% of semi-sweet red wines). Although the type of wine varied in quantity, there could be a subtle difference between concentration, occurrence of Bas, and the winemaking process. In particular, this was most evident between dry red wines and dry white wines. For example, HIS and CAD in dry red wines had higher concentration and more occurrence than that in their white counterparts.

Furthermore, the effects of the storage time, the grape variety, the wine types, and the basic physicochemical indexes (pH, alcoholic degree and residual sugar) of the 81 wine samples on the content of BAs were analyzed using Pearson correlation analysis (Table 4). The results showed that, among the seven kinds of BAs, the concentration of HIS had a certain correlation with alcoholic degree (*p* < 0.01) and grape variety (*p* < 0.05). In addition, the level of PEA had a certain correlation with the wine pH (*p* < 0.01) and wine storage time (*p* < 0.05). In addition, the concentration of PEA was found to be related to the amount of CAD and HEX (*p* < 0.01). The content of TYR in wine was correlated with the presence of HIS (*p* < 0.01) and CAD (*p* < 0.05). Meanwhile, the total concentration of BAs in wine was significantly positively correlated with that of HIS, PUT and TYR (*p* < 0.01), namely, the total amount of BAs in wine is mainly determined by the concentration level of HIS, PUT, and TYR. This is consistent with the rule previously discovered regarding the number of various types of BAs in wine, as well as the research results obtained by Marques et al. [24] and García-Marino et al. [25]. Furthermore, this result also explained why these substances were the most important and more frequently detected BAs in wines. This is caused not only by the high levels of these individual BAs but is also due to the factors that other BA counterparts or some wine parameters may act as potentiators to influence the concentration and toxicity of them. Therefore, the high levels of the individual BA, and the extreme high resulting cumulative concentrations of BAs in wine samples definitely pose a risk for the consumer. 

## 4. Discussion

At present, various methods have been developed for the analysis of BAs in foods. Initially, spectrofluorimetric techniques were used to determine BAs. More recently, chromatographic methods are the techniques most commonly applied to analyze several BAs simultaneously [26]. Owing to the absence of chromophores from these compounds, it is necessary to form derivatives by dansyl chloride (Dns-Cl), dabsyl chloride (DabsCl), and o-phthalaldeyde (OPA) [14]. Lately, there has been a growing interest in the use of electrospray ionization-mass spectrometry (ESI-MS). ESI provides an effective means for ionizing analytes directly from solution prior to their MS analysis without a previous derivatization treatment [3]. The analytical techniques also concerned the molecular methods for the detection of BA producing bacteria. These methods are based on the polymerase chain reaction or PCR technique to detect bacterial amino acid decarboxylases in a rapid, sensitive, and accurate way [27]. Considering that the food matrix is very complex, appropriate extraction and purification methods should be adopted in the sample pretreatment. QuEChERS-based purification technology has been widely used in the pretreatment of complex samples with a number of advantages, such as simplicity, minimum steps, and effectiveness in cleaning-up complex samples [28]. In this experiment, it can be seen in Table 2 that the target BAs showed average recovery values in the range of 77.18% to 101.69%, which indicated that the proposed QuEChERS method coupled with UHPLC-QqQ-MS/MS analysis was sufficiently accurate for the determination of the analyte concentrations in the wine samples.

Meanwhile, no laws or regulations on the dose of BAs in alcoholic beverages have been enacted in China. However, researchers have still proposed the toxic dose of some BAs in alcoholic beverages. According to the standard [3], the BAs in some wines may result in a health hazard. Of course, it must be pointed out that the toxicity threshold of BAs in matrix is also related to the varieties of food [3]. The sensitivity levels of different individuals, when they react to the toxicity of BAs, are significantly different [8]. Therefore, detailed studies still need to be conducted to find out the degree to which the BAs in wine would affect Chinese consumers and how the limit standards should be set.

There are three possible sources of BAs in wine: already existing in the grape musts; formed by yeasts during alcoholic fermentation; or produced by the decarboxylation action of lactic acid bacteria during malolactic fermentation (For example, when the histidine (34 mg/L) was added to the must, the highest HIS values could be obtained from 3.7 to 8.3 mg/L) [26]. Previous research studies showed that BAs have been detected in grapes, and the type and amount of them are affected by factors such as variety, place of origin, year, climate, soil, cultivation, and grade of maturity [29,30,31]. Other factors affecting BA content are the degrees of vintage of grapes as well as irrigation [31]. In addition, Liu et al. [32] studied the effect of cluster thinning on the concentration of BAs in Cabernet Sauvignon grapes and found that the quality of berries that undergone 1.0 cluster/shoot was good, and the amount of BAs in the wine was low. Of course, the control of the amount of BAs in wine does not only involve the production of the raw materials but also concerns the conditions of production and storage. The content of amino acids in grape juice can be adjusted during the vinification process, which includes must adjustment [32], maceration [33], and pumping-over circulation [30], thereby the concentration of BAs can be changed. In addition, the type and amount of BAs in wine can be affected by fermentation temperature, SO_2_ additive amount, pH, alcoholic degree, and dissolved oxygen content [34]. Furthermore, in the process of aging and bottle storage, BAs are also produced in wine [35,36].

Today’s lifestyle and global markets have led to a large consumption of wine, with the new production and protection systems and the development of complex food chains, which in many cases requires a deeper understanding of how these wines are handled and forces us to face the new issues and challenges in providing safe products. Therefore, the safety of wine can be ensured by selecting grapes of good quality and the certain of sanitary conditions at the production stage, thus avoiding the contamination of infectious microbe from producing BAs in the fermentation process, inoculating excellent yeast strains and lactic acid bacteria strains without the activity of amino acid decarboxylase, and adopting appropriate brewing technology and reasonable storage environment. Although it is currently difficult to solve some of these problems, they should be studied and incorporated into future regulations to ensure wine safety and consumer health.

## 5. Conclusions

A reliable and fast method to determine and quantify seven kinds of BAs in wine matrices has been developed and validated in this study. The optimization of MS/MS parameter, UHPLC separation, and QuEChERS procedure, which includes matrix pH condition, solvent, salt type and amount, clean-up agent, were performed, and the whole separation of BAs was accomplished within 6.3 min of analyzing time. Under optimum conditions, linear range, LOD, LOQ, precision, and recovery are good performance and quality results in terms of trueness, reproducibility, and repeatability. This validated method was applied in a pilot study to analyze BAs in 81 wine samples from the Hexi Corridor Region, CAD, HEX, HIS, PEA, PUT, and TYR were detected to varying degrees in the samples. Pearson correlation analysis showed that, among the seven kinds of BAs, the concentration of HIS had a certain correlation with alcoholic degree and grape variety. In addition, the level of PEA had a certain correlation with the wine pH and wine storage time. It was also noticed that the levels of HIS, PUT, and TYR strongly influenced the amine contents of wine. However, when compared with the existing standard, all of the BAs in the 81 kinds of wines did not exceed the prescribed limit value or toxic dose. That is to say, the food safety risks of BAs to wines in the Hexi Corridor Region are relatively low. However, the routine monitoring to BAs is still necessary to guarantee the wine safety in this area [37,38]. 

## Figures and Tables

**Table 1:** MS parameters of seven BAs and the internal standard **^a^**.

Compounds	Abbr	Precursor Ion (*m/z*)	Product Ion ^b^ (*m*/*z*)	Fragmentor (V)	Collision Energy (V)
Cadaverine	CAD	103.0	86.2/41.2	50	5/22
Putrescine	PUT	89.1	72.2/30.2	45	5/20
Histamine	HIS	111.9	95.0/68.2	50	24/12
1,7-diaminoheptane	DAH	131.0	113.9/55.2	70	10/20
Tyramine	TYR	137.9	120.9/77.1	45	5/30
Phenylethylamine	PEA	121.9	104.9/77.1	50	10/30
Hexylamine	HEX	102.0	43.3/41.2	50	15/28
Tryptamine	TRY	160.9	143.8/114.9	55	40/8

^a^ MS: Mass spectrometry; BAs: Biogenic amines. ^b^ Quantifying ions indicated in bold.

**Table 2:** LODs, LOQs, linearity, precision and recovery of seven BAs ^a^.

Compounds ^b^	LOD ^b^ (µg/L)	LOQ (µg/L)	Linearity			Precision (RSD %) ^c^		Recovery (%)		
			Calibration Equations	*R* ^2^	Range (μg/L)	Intra-day (*n* = 6)	Inter-day (*n* = 6)	50 (μg/L)	100 (μg/L)	500 (μg/L)
CAD	0.67	2.21	Y = 0.0072X + 0.1485	0.9976	0.5–800	2.72–6.24	1.13–9.74	83.03 ± 9.49	79.41 ± 6.87	83.23 ± 7.81
PUT	0.67	2.21	Y = 0.0176X + 0.6442	0.9903	0.5–800	2.41–4.45	3.34–4.81	80.34 ± 2.50	93.71 ± 4.88	91.61 ± 6.30
HIS	1.00	3.30	Y = 0.0421X + 0.0967	0.9988	0.5–800	3.03–3.81	2.31–4.53	96.38 ± 0.70	77.18 ± 7.61	90.58 ± 9.73
TYR	0.75	2.50	Y = 0.0682X − 0.1523	0.9991	0.5–800	3.30–4.64	3.26–5.27	91.69 ± 11.42	88.96 ± 8.07	92.23 ± 13.65
PEA	0.50	1.65	Y = 0.1307X + 0.1676	0.9991	0.5–800	2.82–3.36	1.95–2.62	97.67 ± 7.32	86.87 ± 10.06	99.80 ± 15.40
HEX	1.00	3.30	Y = 0.0636X − 0.2141	0.9996	0.5–800	3.69–8.88	4.99–6.29	101.69 ± 12.03	95.96 ± 16.55	101.17 ± 10.13
TRY	0.75	2.50	Y = 0.0491X + 0.0345	0.9990	0.5–800	0.00–7.04	0.00–3.49	93.23 ± 10.38	90.21 ± 7.76	93.52 ± 13.86

^a^ LODs: Limit of detections; LOQs: Limit of quantifications. ^b^ CAD: Cadaverine; PUT: Putrescine; HIS: Histamine; TYR: Tyramine; PEA: Phenylethylamine; HEX: Hexylamine; TRY: Tryptamine. ^c^ RSD: Relative standard deviation.

**Table 3:** BA occurrence in different types of wine samples.

Type of Wine		CAD ^b^	PUT	HIS	TYR	PEA	HEX	TRY ^a^
Dry Red Wine	Number of positive samples/analyzed	57/62	62/62	60/62	62/62	62/62	35/62	-
	Mean (range) µg/L	238.36 (15.03–1337.65)	3525.21 (405.91–10787.44)	931.12 (4.24–4704.05)	335.76 (14.65–2807.30)	448.02 (9.45–2378.34)	26.77 (17.68–59.47)	-
White Ice Wine	Number of positive samples/analyzed	5/6	6/6	3/6	6/6	6/6	3/6	-
	Mean (range) µg/L	275.97 (21.34–448.73)	2319.46 (967.79–4780.97)	733.86 (2.82–2173.29)	470.49 (14.29–2725.91)	633.86 (214.96–1179.09)	26.60 (20.88–37.46)	-
Red Ice Wine	Number of positive samples/analyzed	6/6	6/6	6/6	6/6	6/6	3/6	-
	Mean (range) µg/L	380.84 (21.97–1632.95)	3032.69 (295.04–6083.07)	353.50 (5.33–1942.15)	249.25 (12.86–1369.54)	487.74 (126.36–905.96)	35.68 (27.55–50.25)	-
Dry White Wine	Number of positive samples/analyzed	2/3	3/3	2/3	3/3	3/3	1/3	-
	Mean (range) µg/L	48.54 (25.52–71.55)	2227.71 (1627.65–2530.45)	22.66 (10.61–34.71)	15.68 (12.76–19.62)	202.42 (127.49–343.69)	30.39	-
Sweet Red Wine	Number of positive samples/analyzed	2/3	3/3	1/3	3/3	3/3	2/3	-
	Mean (range) µg/L	86.95 (70.86–103.04)	2647.07 (999.86–5074.44)	1046.44	45.73 (12.82–104.44)	498.58 (12.71–937.60)	19.32 (18.24–20.43)	-
Semi-Sweet Red Wine	Number of positive samples/analyzed	1/1	1/1	1/1	1/1	1/1	1/1	-
	Mean (range) µg/L	67.21	1832.62	36.68	19.52	135.75	23.83	-

^a^ means not detected; ^b^ CAD: Cadaverine; PUT: Putrescine; HIS: Histamine; TYR: Tyramine; PEA: Phenylethylamine; HEX: Hexylamine; TRY: Tryptamine.

**Table 4:** Correlation analysis between BAs and wine parameters.

	Type of Wine ^a^	Grape Variety	Storage Time	pH	Alcoholic Degree	Residual Sugar	PUT ^b^	HEX	CAD	HIS	PEA	TYR	Total BAs
Type of Wine	1												
Grape Variety	−0.052	1											
Storage Time	0.219 *	0.022	1										
pH	−0.397 **	−0.179	−0.347 **	1									
Alcoholic Degree	−0.058	0.341 **	−0.068	0.299 **	1								
Residual Sugar	−0.035	0.053	0.075	0.190	0.172	1							
PUT	−0.030	0.148	−0.167	−0.058	0.116	−0.199	1						
HEX	−0.010	0.022	0.080	0.120	0.019	0.133	0.009	1					
CAD	−0.158	0.098	−0.046	0.046	−0.125	0.053	0.041	0.246 *	1				
HIS	0.084	0.236 *	−0.133	−0.026	0.417 **	−0.148	0.176	0.006	0.024	1			
PEA	0.282 *	0.026	0.258 *	−0.287 **	−0.080	0.131	0.043	0.350 **	0.310 **	0.042	1		
TYR	−0.052	−0.004	0.038	−0.188	−0.143	−0.006	−0.066	0.023	0.228 *	0.476 **	0.205	1	
Total BAs	0.026	0.214	−0.131	−0.134	0.191	−0.179	0.797 **	0.097	0.242 *	0.640 **	0.280 *	0.423 **	1

^a^ * and ** indicate significant differences (confidence, double test) at *p* < 0.05 and *p* < 0.01, respectively; ^b^ CAD: Cadaverine; PUT: Putrescine; HIS: Histamine; TYR: Tyramine; PEA: Phenylethylamine; HEX: Hexylamine; TRY: Tryptamine.

## Supplementary Materials

The following are available online at https://www.mdpi.com/2304-8158/8/11/552/s1, Table S1. Individual BA and wine parameters in 81 wine samples from Hexi Corridor Region (*n* = 3).

## References

[1] Omanovic-Miklicanin E., Valzacchi S. (2017). Development of new chemiluminescence biosensors for determination of biogenic amines in meat. Food Chem..

[2] Mattsson L., Xu J., Preininger C., Tse Sum Bui B., Haupt K. (2018). Competitive fluorescent pseudo-immunoassay exploiting molecularly imprinted polymers for the detection of biogenic amines in fish matrix. Talanta.

[3] Moreno-Arribas M.V., Polo M.C. (2009). Wine Chemistry and Biochemistry.

[4] Linares D.M., Martín M., Ladero V., Alvarez M.A., Fernández M. (2011). Biogenic amines in dairy products. Crit. Rev. Food Sci. Nutr..

[5] Vidal-Carou M.C., Veciana-Nogués M.T., Latorre-Moratala M.L., Bover-Cid S. (2008). Biogenic Amines: Risks and Control. Handbook of Fermented Meat and Poultry.

[6] Kim S.H., Eun J.B., Chen T.Y., Wei C.I., Clemens R.A., An H. (2010). Evaluation of histamine and other biogenic amines and bacterial isolation in canned anchovies recalled by the USFDA. J. Food Sci..

[7] Costa M.P., Rodrigues B.L., Frasao B.S., Conte-Junior C.A., Holban A.M., Grumezescu A.M. (2018). Chapter 2-Biogenic Amines as Food Quality Index and Chemical Risk for Human Consumption. Food Quality: Balancing Health and Disease.

[8] Pérez-Rodríguez F., Skandamis P., Valdramidis V. (2018). Quantitative Methods for Food Safety and Quality in the Vegetable Industry.

[9] Ladero V., Calles-Enriquez M., Fernandez M., Alvarez M.A. (2010). Toxicological effects of dietary biogenic amines. Curr. Nutr. Food Sci..

[10] Hong J.Y., Na H.P., Oh M.S., Lee H.S., Pyo H., Hong J. (2013). Profiling analysis of biogenic amines and their acidic metabolites in mouse brain tissue using gas chromatography–tandem mass spectrometry. J. Chromatogr. B.

[11] Ščavničar A., Rogelj I., Kočar D., Kӧse S., Pompe M. (2018). Determination of biogenic amines in cheese by ion chromatography with tandem mass spectrometry detection. J. AOAC Int..

[12] Marcobal A., Polo M.C., Martín-Álvarez P.J., Moreno-Arribas M.V. (2005). Biogenic amine content of red Spanish wines: Comparison of a direct ELISA and an HPLC method for the determination of histamine in wines. Food Res. Int..

[13] Xu J.J., Ma C.L., Guo C.F. (2018). A comparative analysis of derivatization strategies for the determination of biogenic amines in sausage and cheese by HPLC. Food Chem..

[14] Wang Y.Q., Ye D.Q., Zhu B.Q., Wu G.F., Duan C.Q. (2014). Rapid HPLC analysis of amino acids and biogenic amines in wines during fermentation and evaluation of matrix effect. Food Chem..

[15] Manetta A.C., Giuseppe L.D., Tofalo R., Martuscelli M., Schirone M., Giammarco M., Suzzi G. (2016). Evaluation of biogenic amines in wine: Determination by an improved HPLC-PDA method. Food Control.

[16] Jain A., Gupta M., Verma K.K. (2015). Salting-out assisted liquid–liquid extraction for the determination of biogenic amines in fruit juices and alcoholic beverages after derivatization with 1-naphthylisothiocyanate and high performance liquid chromatography. J. Chromatogr. A.

[17] Huang K.J., Wei C.Y., Liu W.L., Xie W.Z., Zhang J.F., Wang W. (2009). Ultrasound-assisted dispersive liquid–liquid microextraction combined with high-performance liquid chromatography-fluorescence detection for sensitive determination of biogenic amines in rice wine samples. J. Chromatogr. A.

[18] Fernández-Franzón M., Berardinis F.D., Sagratini G., Vittori S., Font G., Mañes J. (2010). Simultaneous determination of eight biogenic amines in fish by liquid chromatography with fluorescence detector and tandem mass spectrometry. Toxicol. Lett..

[19] Papageorgiou M., Lambropoulou D., Morrison C., Kłodzińska E., Namieśnik J., Płotka-Wasylka J. (2018). Literature update of analytical methods for biogenic amines determination in food and beverages. Trends Anal. Chem..

[20] Dong H., Xiao K. (2017). Modified QuEChERS combined with ultra high performance liquid chromatography tandem mass spectrometry to determine seven biogenic amines in Chinese traditional condiment soy sauce. Food Chem..

[21] Lozanov V., Benkova B., Mateva L., Petrov S., Popov E., Slavov C., Mitev V. (2007). Liquid chromatography method for simultaneous analysis of amino acids and biogenic amines in biological fluids with simultaneous gradient of pH and acetonitrile. J. Chromatogr. B.

[22] Cai Y., Sun Z., Chen G., Liu X., You J., Zhang C. (2016). Rapid analysis of biogenic amines from rice wine with isotope-coded derivatization followed by high performance liquid chromatography-tandem mass spectrometry. Food Chem..

[23] Cong L., Huang B., Chen Q., Lu B., Zhang J., Ren Y. (2006). Determination of trace amount of microcystins in water samples using liquid chromatography coupled with triple quadrupole mass spectrometry. Anal. Chim. Acta.

[24] Ramos R.M., Valente I.M., Rodrigues J.A. (2014). Analysis of biogenic amines in wines by salting-out assisted liquid–liquid extraction and high-performance liquid chromatography with fluorimetric detection. Talanta.

[25] Marques A.P., Leitão M.C., San Romão M.V. (2008). Biogenic amines in wines: Influence of oenological factors. Food Chem..

[26] García-Marino M., Trigueros Á., Escribano-Bailón T. (2010). Influence of oenological practices on the formation of biogenic amines in quality red wines. J. Food Compos. Anal..

[27] Peña-Gallego A., Hernández-Orte P., Cacho J., Ferreira V. (2009). Biogenic amine determination in wines using solid-phase extraction: A comparative study. J. Chromatogr. A.

[28] Lucas P.M., Olivier C., Aline L.F. (2008). High frequency of histamine-producing bacteria in the enological environment and instability of the histidine decarboxylase production phenotype. Appl. Environ. Microbiol..

[29] Jovanov P., Guzsvány V., Franko M., Lazić S., Sakač M., Milovanović I., Nedeljković N. (2014). Development of multiresidue DLLME and QuEChERS based LC–MS/MS method for determination of selected neonicotinoid insecticides in honey liqueur. Food Res. Int..

[30] Prete V.D., Costantini A., Cecchini F., Morassut M., Garcia-Moruno E. (2009). Occurrence of biogenic amines in wine: The role of grapes. Food Chem..

[31] Cecchini F., Morassut M. (2010). Effect of grape storage time on biogenic amines content in must. Food Chem..

[32] Ortega-Heras M., Pérez-Magariño S., Del-Villar-Garrachón V., González-Huerta C., Moro Gonzalez L.C., Guadarrama R.A., Villanueva S.S., Gallo G.R., Martín D.L.H.S. (2014). Study of the effect of vintage, maturity degree, and irrigation on the amino acid and biogenic amine content of a white wine from Verdejo variety. J. Sci. Food Agric..

[33] Liu P., Wang Y., Wu G. (2015). Effect of cluster thinning on concentration of biogenic amines in Cabernet sauvignon grape berries and wines. J. Chin. Inst. Food Sci. Technol..

[34] Sacchi K.L., Bisson L.F., Adams D.O. (2005). A review of the effect of winemaking techniques on phenolic extraction in red wines. Am. J. Enol. Vitic..

[35] Soleas G.J., Carey M., Goldberg D.M. (1999). Method development and cultivar-related differences of nine biogenic amines in Ontario wines. Food Chem..

[36] Lorenzo C., Bordiga M., Pérez-Álvarez E.P., Travaglia F., Arlorio M., Salinas M.R., Coïsson J.D., Garde-Cerdán T. (2017). The impacts of temperature, alcoholic degree and amino acids content on biogenic amines and their precursor amino acids content in red wine. Food Res. Int..

[37] Ordóñez J.L., Callejón R.M., Troncoso A.M., García–Parrilla M.C. (2017). Evaluation of biogenic amines profile in opened wine bottles: Effect of storage conditions. J. Food Compos. Anal..

[38] Bach B., Le Quere S., Vuchot P., Grinbaum M., Barnavon L. (2012). Validation of a method for the analysis of biogenic amines: Histamine instability during wine sample storage. Anal. Chim. Acta.

