# Supplementary material for: Development and Application of a New QuEChERS Method in UHPLC-QqQ-MS/MS to Detect Seven Biogenic Amines in Chinese Wines
Source: Foods. 2019 Nov 5;8(11):552. doi: 10.3390/foods8110552 (PMC6915522; doi:10.3390/foods8110552)
Supplement: Supplementary file 1 [file foods-08-00552-s001.pdf]

Supplementary Material:

Development and application of a new QuEChERS method in UHPLC-QqQ-MS/MS to detect seven biogenic amines in Chinese wines

Shun-Yu Han, Lan-Lan Hao, Xiao Shi, Jian-Ming Niu and Bo Zhang *

Gansu Key Laboratory of Viticulture and Enology, College of Food Science and Engineering, Gansu Agricultural University, Lanzhou 730070, China

* Corresponding author.
Address: No. 1, Yingmen Town, Anning District, Lanzhou City, Gansu Province, 730070, PR China.
Tel.: +86-0931-7631-201.
Fax: +86-0931-7631-201.
E-mail: zhangbo@gsau.edu.cn.

Table S1. Individual BA and wine patameters in 81 wine samples from Hexi Corridor Region (n = 3).

No.	Type	Grape Variety	Year	pH	Alcohol (%)	Residual Sugar (g/L)	CAD [a, b] (μg/L)	PUT (μg/L)	HIS (μg/L)	TYR (μg/L)	PEA (μg/L)	HEX (μg/L)	TRY (μg/L)	Total BAs (μg/L)
1	Dry Red Wine	Cabernet Sauvignon	2008	3.55	12.06	1.30	-	3237.46±242.64	4.24±0.32	17.25±0.78	250.54±4.25	-	-	3509.49
2	White Ice Wine	Pinot Blanc+Riesling	2008	3.66	11.95	64.30	-	1502.68±88.48	-	18.31±0.24	214.96±16.73	-	-	1735.95
3	Red Ice Wine	Merlot	2008	3.74	11.99	64.20	32.17±10.59	6083.07±243.34	6.02±0.85	19.37±1.1	608.60±54.55	-	-	6749.23
4	Sweet Red Wine	Blended	2008	3.44	7.77	52.10	70.86±16.26	1866.90±97.25	-	19.93±0.58	937.60±31.02	-	-	2895.29
5	Dry White Wine	Italian Riesling	2008	3.18	11.79	2.80	-	2525.03±48.14	-	12.76±0.02	127.49±10.4	-	-	2665.28
6	White Ice Wine	Pinot Blanc+Riesling	2009	3.53	12.46	76.40	21.34±3.08	2076.67±66.92	-	14.29±0.16	293.33±41.04	-	-	2405.63
7	Dry Red Wine	Pinot Noir	2009	3.69	12.33	1.70	95.23±6.16	4099.88±150.66	-	19.19±0.71	1008.89±35.03	28.42±3.17	-	5251.61
8	Dry White Wine	Italian Riesling	2009	3.45	12.16	1.40	25.52±1.48	2530.45±91.54	10.61±5.07	14.65±0.23	343.69±25.98	30.39±2.65	-	2955.31
9	Red Ice Wine	Merlot	2009	3.43	11.02	60.00	1632.95±143.26	4801.86±174.23	1942.15±35.08	1369.54±88.21	905.96±30.45	27.55±1.56	-	10680.01
10	Dry Red Wine	Pinot Noir	2009	3.62	11.96	2.00	551.81±33.22	1602.18±89.56	13.16±1.53	38.01±2.74	578.91±59.13	27.78±0.33	-	2811.85
11	White Ice Wine	Pinot Blanc+Riesling	2009	3.37	11.42	90.70	63.07±4.37	2538.74±290.4	25.47±3.74	29.43±0.55	705.91±39.09	20.88±0.71	-	3383.50
12	Dry Red Wine	Cabernet Sauvignon	2009	3.93	13.64	1.70	15.03±10.47	1187.79±68.08	1654.82±40.21	1444.41±104.65	118.29±13.78	29.61±2.80	-	4449.95
13	Dry Red Wine	Blended	2010	3.53	11.71	1.30	41.90±3.4	1404.60±113.23	-	18.39±0.66	497.00±41.4	20.84±0.26	-	1982.73
14	Dry Red Wine	Pinot Noir	2010	3.71	12.35	2.10	321.57±14.69	3986.60±226.13	88.10±4.17	39.60±1.58	2089.63±102.25	59.47±58.49	-	6584.97
15	Dry Red Wine	Cabernet Sauvignon	2010	3.93	14.33	1.80	138.50±16.47	1784.90±70.77	3020.13±26.31	1985.90±138.12	185.38±23.96	-	-	7114.81
16	Dry Red Wine	Blended	2011	3.55	12.03	1.60	133.43±9.63	3200.01±76.43	67.30±4.94	1081.82±95.77	1501.41±107.75	24.45±0.52	-	6008.42
17	Dry Red Wine	Blended	2012	3.52	11.79	2.10	288.73±34.47	3066.34±262.88	2066.91±105.06	654.61±13.25	2378.34±115.15	28.35±0.56	-	8483.28
18	Dry Red Wine	Cabernet Sauvignon	2012	3.98	13.59	1.70	118.59±6.55	3580.55±122.88	523.36±5.57	34.99±1.28	191.97±21.23	23.49±1.59	-	4472.95
19	Dry Red Wine	Pinot Noir	2013	3.58	12.41	2.30	210.49±28.55	2488.52±291.97	1723.78±157.85	1741.36±42.17	1424.36±102.77	25.45±1.83	-	7613.96
20	Dry Red Wine	Cabernet Sauvignon	2013	3.88	13.31	1.70	63.57±7.10	1524.76±23.57	1446.10±33.35	1211.49±35.11	135.05±26.21	-	-	4380.97

Table S1. *Cont.*

No.	Type	Grape Variety	Year	pH	Alcohol (%)	Residual Sugar (g/L)	CAD (μg/L)	PUT (μg/L)	HIS (μg/L)	TYR (μg/L)	PEA (μg/L)	HEX (μg/L)	TRY (μg/L)	Total BAs (μg/L)
21	Dry Red Wine	Cabernet Sauvignon	2013	4.16	16.95	2.40	90.75±14.52	682.81±37.89	21.79±1.81	14.65±0.11	44.11±3.60	19.20±0.30	-	873.31
22	Dry Red Wine	Blended	2014	3.59	12.41	2.10	292.62±20.79	3548.73±211.64	1362.36±42.4	1073.84±51.45	1055.74±62.14	24.98±0.75	-	7358.27
23	Dry Red Wine	Merlot	2014	3.95	13.58	1.8	-	3983.47±154.84	3241.95±124.78	19.76±0.27	176.06±5.25	28.83±1.61	-	7450.07
24	Dry Red Wine	Merlot	2014	3.74	13.01	1.5	266.62±24.96	3580.45±234.21	719.68±18.92	31.58±5.72	913.49±158.06	-	-	5511.82
25	Dry Red Wine	Cabernet Sauvignon	2014	3.84	13.94	1.80	170.31±21.22	3986.16±102.22	31.16±0.15	19.13±0.33	127.83±12.27	-	-	4334.59
26	Dry White Wine	Italian Riesling	2014	3.63	12.88	1.2	71.55±2.79	1627.65±135.67	34.71±3.56	19.62±0.28	136.07±5.66	-	-	1889.6
27	Dry Red Wine	Pinot Noir	2014	4.48	15.38	2.20	771.96±8.20	2048.49±42.30	108.76±3.50	21.54±0.40	309.98±5.32	27.92±0.38	-	3288.65
28	Dry Red Wine	Cabernet Sauvignon+Merlot	2014+2017	3.86	13.58	2.10	30.62±3.27	4157.65±92.98	29.36±0.83	19.77±0.82	150.61±4.26	-	-	4388.01
29	Dry Red Wine	Blended	2015	3.50	11.59	2.10	76.04±22.15	2439.56±267.1	2681.36±258.86	1650.58±16.51	651.32±56.21	22.93±0.83	-	7521.79
30	White Ice Wine	Italian Riesling	2015	4.81	8.16	278.4	448.73±11.19	967.79±33.35	2.82±0.15	14.44±0.21	1112.32±72.66	37.46±0.09	-	2583.56
31	Dry Red Wine	Cabernet Sauvignon	2015	4.10	15.42	1.90	298.91±8.32	2484.44±54.92	58.29±1.74	19.65±0.72	119.96±3.48	25.23±0.77	-	3006.48
32	Dry Red Wine	Cabernet Gernischt	2015	3.69	11.69	4.60	380.97±19.37	2282.61±180.22	128.93±12.64	53.85±4.47	398.61±40.2	-	-	3244.97
33	Dry Red Wine	Cabernet Sauvignon	2015	3.86	13.48	1.50	50.80±11.05	2120.91±48.17	15.49±0.59	16.77±0.50	113.48±7.10	-	-	2317.45
34	Dry Red Wine	Cabernet Gernischt	2015+2016	3.99	13.78	1.5	18.47±15.47	7557.96±621.28	3292.77±333.61	19.82±1.16	239.63±21.01	42.03±2.09	-	11170.68
35	Dry Red Wine	Cabernet Sauvignon+Merlot	2015+2016	3.86	13.25	1.80	81.35±8.41	3711.03±192.79	22.46±0.58	22.77±0.93	182.75±16.73	33.72±2.79	-	4054.08
36	Dry Red Wine	Merlot	2015+2016	3.86	13.24	1.70	68.76±6.28	3581.76±99.84	25.43±0.83	22.19±0.78	174.57±11.76	-	-	3872.71
37	Dry Red Wine	Blended	2015+2016	3.77	12.46	1.30	175.95±10.12	6715.92±270.46	19.29±0.92	35.35±0.80	744.06±45.30	22.48±1.02	-	7713.05
38	Dry Red Wine	Pinot Noir	2015+2016	3.99	13.61	1.40	1074.59±50.81	2858.41±142.32	43.70±1.54	40.08±1.13	1061.38±22.72	24.32±0.21	-	5102.48
39	Dry Red Wine	Merlot	2015+2016+2017	3.88	13.40	1.80	101.35±20.15	3334.14±227.56	187.07±4.51	26.34±0.60	302.03±28.78	36.05±1.01	-	3986.98

Table S1. *Cont.*

No.	Type	Grape Variety	Year	pH	Alcohol (%)	Residual Sugar (g/L)	CAD (μg/L)	PUT (μg/L)	HIS (μg/L)	TYR (μg/L)	PEA (μg/L)	HEX (μg/L)	TRY (μg/L)	Total BAs (μg/L)
40	Dry Red Wine	Cabernet Sauvignon	2015+2017	4.01	14.07	2.30	43.39±5.96	7662.16±217.58	297.19±5.69	18.44±0.16	155.24±5.27	-	-	8176.42
41	Dry Red Wine	Cabernet Sauvignon	2016	4.06	14.73	2.10	-	1574.50±2.22	13.20±0.12	16.66±0.25	104.25±2.84	-	-	1708.61
42	Dry Red Wine	Blended	2016	3.84	12.95	1.80	274.56±15.03	3563.58±91.86	63.85±0.48	47.38±2.08	891.33±30.45	-	-	4840.70
43	Dry Red Wine	Cabernet Sauvignon	2016	4.17	15.36	2.30	58.10±19.67	4432.62±71.06	23.84±0.71	20.91±0.61	134.86±1.57	-	-	4670.33
44	Dry Red Wine	Merlot	2016	3.56	11.80	1.90	302.44±21.54	4006.25±164.36	3114.12±36.9	132.93±3.2	604.75±60.28	22.15±0.88	-	8182.64
45	Dry Red Wine	Cabernet Sauvignon	2016	4.05	15.60	2.40	203.67±38.67	1975.67±220.15	43.68±1.07	15.36±0.50	116.73±12.38	23.79±1.47	-	2378.90
46	Dry Red Wine	Cabernet Sauvignon	2016	4.09	15.17	1.80	271.03±5.14	2365.98±24.37	44.29±0.65	15.69±0.25	109.13±4.55	21.76±0.59	-	2827.88
47	Dry Red Wine	Cabernet Sauvignon	2016	4.10	15.35	2.90	378.30±21.75	2594.89±111.73	41.63±2.68	16.05±0.25	113.68±2.53	22.77±0.49	-	3167.32
48	Dry Red Wine	Merlot	2016	4.10	12.54	2.10	1337.65±74.17	4838.15±293.01	68.37±11.68	31.16±0.65	327.88±26.97	31.57±3.99	-	6634.78
49	Dry Red Wine	Pinot Noir	2016	3.59	12.27	1.80	309.77±40.55	1847.81±170.97	1906.04±125.47	1582.21±33.5	519.49±26.91	22.12±1.16	-	6187.44
50	Dry Red Wine	Cabernet Sauvignon	2016+2017	3.87	13.44	1.60	-	3055.06±20.96	13.89±0.66	18.50±0.50	105.40±7.62	23.89±0.41	-	3216.74
51	Dry Red Wine	Blended	2016+2017	3.92	13.49	1.90	287.08±30.33	3605.47±55.44	15.16±0.51	22.26±0.21	391.65±7.92	-	-	4321.62
52	Dry Red Wine	Cabernet Sauvignon	2016+2017	3.69	12.87	1.40	226.17±14.26	3241.47±134.42	121.15±0.95	56.73±2.84	295.35±17.5	-	-	3940.87
53	Red Ice Wine	Merlot	2017	3.44	10.91	72.90	43.25±1.86	2892.57±108.13	42.38±0.98	26.02±1.72	434.97±64.94	-	-	3439.19
54	Dry Red Wine	Blended	2017	3.57	11.58	2.00	231.65±8.89	7115.94±207.59	2360.78±115.09	95.24±5.99	491.33±8.73	22.16±0.54	-	10317.10
55	Sweet Red Wine	Blended	2017	3.36	8.27	50.40	103.04±7.31	5074.44±102.05	1046.44±34.98	104.44±18.7	545.42±17.16	20.43±0.65	-	6894.21
56	Dry Red Wine	Pinot Noir	2017	3.68	12.53	2.50	91.38±15.73	9401.57±126.92	1637.54±77.46	925.56±62.92	417.60±20.66	19.90±0.69	-	12493.55
57	White Ice Wine	Pinot Blanc+Riesling	2017	3.62	11.54	95.80	430.35±25.09	2049.91±198.74	-	20.57±0.68	1179.09±47.11	21.45±1.35	-	3701.37
58	Dry Red Wine	Pinot Noir	2017	3.92	11.45	0.70	24.19±3.00	8336.42±306.75	553.39±27.98	529.85±50.12	138.17±10.19	17.68±0.11	-	9599.7
59	Red Ice Wine	Merlot	2017	3.37	12.75	2.60	512.5±13.11	3398.42±67.51	74.62±2.25	39.52±2.40	523.20±35.95	-	-	4548.26
60	Red Ice Wine	Blended	2017	3.53	11.11	63.70	21.97±4.34	295.04±21.70	5.33±0.56	28.23±0.24	327.32±24.25	50.25±1.04	-	728.14

Table S1. *Cont.*

No.	Type	Grape Variety	Year	pH	Alcohol (%)	Residual Sugar (g/L)	CAD (μg/L)	PUT (μg/L)	HIS (μg/L)	TYR (μg/L)	PEA (μg/L)	HEX (μg/L)	TRY (μg/L)	Total BAs (μg/L)
61	Dry Red Wine	Cabernet Franc	2017	3.86	13.82	1.60	17.04±14.55	405.91±7.67	2276.45±89.52	2807.3±95.49	396.42±16.16	-	-	5903.12
62	Dry Red Wine	Pinot Noir	2017	3.86	14.11	2.60	1215.04±65.66	2905.96±141.21	2550.42±83.81	2589.58±47.75	933.85±45.23	28.06±4.39	-	10222.91
63	Dry Red Wine	Merlot	2017	3.77	13.15	1 .40	185.58±16.84	942.12±109.61	7.86±0.95	21.18±1.10	246.32±14.09	-	-	1403.06
64	White Ice Wine	Pinot Blanc+Riesling	2017	3.47	11.75	108.70	416.36±15.65	4780.97±221.34	2173.29±159.83	2725.91±130.81	297.53±4.69	-	-	10394.06
65	Red Ice Wine	Merlot	2017	4.74	10.61	151.2	42.21±13.97	725.20±20.52	50.50±0.81	12.86±0.16	126.36±2.41	29.23±1.47	-	986.36
66	Dry Red Wine	Merlot	2017	4.11	15.04	3.2	-	1932.57±419.96	4704.05±805.68	17.93±0.53	222.25±27.05	-	-	6876.8
67	Dry Red Wine	Cabernet Gernischt	2017	3.99	12.99	1.2	130.66±3.19	10349.91±348.95	3898.48±233.95	29.74±0.83	118.97±6.97	-	-	14527.76
68	Dry Red Wine	Cabernet Sauvignon	2017	4.15	14.57	2.50	125.76±3.36	3462.78±62.33	51.09±3.69	18.28±0.65	263.49±7.89	23.56±1.03	-	3944.96
69	Dry Red Wine	Cabernet Sauvignon	2017	4.01	14.58	2.80	61.21±1.83	1175.14±45.93	1985.21±85.36	18.36±0.55	9.45±0.26	24.23±1.13	-	3273.60
70	Dry Red Wine	Cabernet Sauvignon	2017	3.99	14.20	2.70	114.48±10.78	1879.43±65.47	2025.93±86.16	17.48±0.36	157.21±5.50	-	-	4194.53
71	Dry Red Wine	Merlot	2017	3.78	13.71	2.60	164.94±14.99	4591.45±208.98	111.91±1.71	22.7±1.48	23.18±1.86	-	-	4914.18
72	Dry Red Wine	Merlot	2017	4.03	14.40	2.70	95.48±27.37	2564.35±464.12	2366.07±367.32	18.28±0.61	179.42±16.51	-	-	5223.60
73	Dry Red Wine	Blended	2017	3.87	12.91	1.20	347.19±22.91	3810.61±147.52	34.80±2.81	26.94±0.34	336.44±22.13	-	-	4555.98
74	Dry Red Wine	Blended	2017	3.98	13.55	1.10	129.57±6.33	10787.44±138.16	60.37±4.13	25.12±0.70	636.25±32.17	20.41±0.16	-	11659.16
75	Dry Red Wine	Cabernet Sauvignon	2017	3.77	13.99	1.70	235.26±33.31	3384.99±177.2	152.25±12.31	53.55±1.73	203.95±32.15	-	-	4030.00
76	Dry Red Wine	Cabernet Sauvignon	2017	3.73	13.51	1.50	339.26±28.13	3266.85±247.95	122.73±10.24	48.06±3.89	389.62±59.15	37.31±9.52	-	4203.83
77	Dry Red Wine	Cabernet Sauvignon	2017	3.75	14.12	1.90	47.36±14.66	2162.93±181.70	1969.69±151.29	37.67±1.63	655.97±12.55	-	-	4873.62
78	Sweet Red Wine	Merlot	2017	3.76	13.63	70.50	-	999.86±12.87	-	12.82±0.07	12.71±0.57	18.24±0.30	-	1043.63
79	Semi-Sweet Red Wine	Merlot	2017	3.96	15.14	33.80	67.21±25.97	1832.60±287.49	36.68±4.62	19.52±1.22	135.75±22.18	23.83±2.27	-	2115.59
80	Dry Red Wine	Merlot	2017	3.78	12.21	3.70	269.85±20.84	1853.63±140.28	576.35±24.81	99.32±7.16	483.55±29.76	-	-	3282.7
81	Dry Red Wine	Cabernet Sauvignon	2017	4.04	15.81	1.80	139.34±26.34	3231.06±249.15	57.48±0.94	25.98±1.62	478.6±31.79	-	-	3932.46

[a] - means not detected; [b] CAD: Cadaverine; PUT: Putrescine; HIS: Histamine; DAH: 1,7-diaminoheptane; TYR: Tyramine; PEA: Phenylethylamine; HEX: Hexylamine; TRY: Tryptamine.
